# The Nuclear 35S rDNA World in Plant Systematics and Evolution: A Primer of Cautions and Common Misconceptions in Cytogenetic Studies

**DOI:** 10.3389/fpls.2022.788911

**Published:** 2022-02-24

**Authors:** Josep A. Rosselló, Alexis J. Maravilla, Marcela Rosato

**Affiliations:** Jardín Botánico, Instituto Cavanilles de Biodiversidad y Biología Evolutiva, Universitat de València, Valencia, Spain

**Keywords:** 35S rDNA, secondary constriction, satellite chromosome, NOR, nucleolus, amphiplasty, cytogenetic markers, rRNA genes

## Abstract

The ubiquitous presence of rRNA genes in nuclear, plastid, and mitochondrial genomes has provided an opportunity to use genomic markers to infer patterns of molecular and organismic evolution as well as to assess systematic issues throughout the tree of life. The number, size, location, and activity of the 35S rDNA cistrons in plant karyotypes have been used as conventional cytogenetic landmarks. Their scrutiny has been useful to infer patterns of chromosomal evolution and the data have been used as a proxy for assessing species discrimination, population differentiation and evolutionary relationships. The correct interpretation of rDNA markers in plant taxonomy and evolution is not free of drawbacks given the complexities derived from the lability of the genetic architecture, the diverse patterns of molecular change, and the fate and evolutionary dynamics of the rDNA units in hybrids and polyploid species. In addition, the terminology used by independent authors is somewhat vague, which often complicates comparisons. To date, no efforts have been reported addressing the potential problems and limitations involved in generating, utilizing, and interpreting the data from the 35S rDNA in cytogenetics. This review discusses the main technical and conceptual limitations of these rDNA markers obtained by cytological and karyological experimental work, in order to clarify biological and evolutionary inferences postulated in a systematic and phylogenetic context. Also, we provide clarification for some ambiguity and misconceptions in terminology usually found in published work that may help to improve the usage of the 35S ribosomal world in plant evolution.

## Introduction

Ribosomes are universal cellular components found across all domains of life. Research suggests they represent the most critical macromolecular machine in living organisms, as they are trusted with carrying out protein synthesis in cells by converting information encoded within mRNA into peptides (Ojha et al., 2020). Ribosome biogenesis in eukaryotes is a process of extraordinary complexity (Thomson et al., 2013). Four rRNA species are transcribed by two RNA polymerases, RNA Pol I (18S, 5.8S, 26S rRNA) and RNA Pol III (5S rRNA) being extensively modified during their subsequent maturation in the macromolecular complex of the nucleolus, the nucleus and the cytoplasm (Sloan et al., 2017).

The ubiquitous presence of rDNA genes in nuclear, plastid and mitochondrial genomes has provided an opportunity to use ribosomal sequences as homologous markers to infer evolutionary processes and to assess systematic issues at the three basic domains of life, Bacteria, Archaea, Eukarya, including plants (Álvarez and Wendel, 2003; Nieto Feliner and Rosselló, 2007). In fact, the 5S intergenic spacers and the internal transcribed spacers (ITS) from the nuclear ribosomal 35S repeat have been proposed as nuclear standards for species identification (Gemeinholzer et al., 2006; Doveri and Lee, 2007; Gao et al., 2010), species delimitation (Müller et al., 2007) and DNA barcoding (Kress et al., 2005; Chase et al., 2007; CBOL Plant Working Group, 2009) in land plant lineages.

Ribosomal markers are not only limited to the use of rDNA sequences. Since the pioneering studies on the topic (Heitz, 1931), there has been extensive karyological work characterizing rDNA (García et al., 2017). The study of the development of the nucleolus, the assessment of karyological landmarks related to the linear differentiation of chromosomes, like non-centromeric constrictions and associated (satellite) regions, and the physical mapping and linkage between 35S rDNA and 5S rDNA families, have provided anchor points for comparative studies (Weiss-Schneeweiss and Schneeweiss, 2013). These include assessing the molecular evolution of rDNA units, gene silencing, evolutionary trends on karyotype differentiation, producing genetic maps and identifying ancestors in hybrids and polyploid species (Weiss-Schneeweiss et al., 2013). These karyo-evolutionary trends have complemented the knowledge on nuclear rDNA using DNA sequencing and are clearly relevant for providing cytogenetic markers in species that are routinely used for plant systematics and evolution purposes, and to postulate phylogenetic hypotheses (Totta et al., 2017).

The number, size, location and transcriptional activity of the 35S rDNA cistrons in plant karyotypes are some of the traditional cytogenetic landmarks reported in conventional karyotype descriptions (Battaglia, 1955). Their scrutiny has been useful to infer patterns of chromosomal evolution and the data have been used as a proxy for species discrimination, population differentiation and relationships at several evolutionary levels (Guerra, 2012). In agreement with their continued use in the field of cytotaxonomy, no major concerns have been reported for their application as cytogenetic markers in plant taxonomy and evolution (García et al., 2017).

The correct interpretation of 35S rDNA markers in plant taxonomy and phylogenetics is not free of drawbacks. This is not surprising given the complexities derived from the lability of the genetic architecture, the patterns of molecular evolution, and the evolutionary dynamics of the rDNA units, including the number and position of rDNA sites in the genome, sequence homogenization, structure and organization of rRNA genes (Feliner and Rosselló, 2012). In addition, the terminology used by independent authors is somewhat vague, which often complicates comparisons.

This complexity does not appear to be fully appreciated by some systematic plant studies that use chromosomal, or cytological-based rDNA markers, alone or in combination with genomic data. Unfortunately, misconceptions and misuses may lead to far-reaching conclusions based on risky assumptions that do not have general value, or on the use of confusing terminology (Tables 1#x2013;4). To date, no efforts acknowledging the potential problems and limitations involved in generating, utilizing, and interpreting the raw ribosomal data have been reported.

**TABLE 1:** The nuclear 35S rDNA landmarks and significant terminology associated to its activity, detection and morphology.

Assumption	Comments	Selected references
Plant species show chromosome complements lacking secondary constrictions	The presence of rDNA genes in the nuclear genome is a requisite for viable cellular metabolism in eukaryotes. Therefore, a minimum number of active ribosomal 35S rDNA units should be present in the nuclear genome. However, recognizing their location by the observed of decondensed chromatin along the chromosome (secondary constriction) may be compromised due to the use of conventional techniques (standard stains) that lack sensitivity, their position at subterminal or terminal ends of the chromosomes and the number of rDNA units and their activity. Alternative, more powerful techniques (Ag-NOR, immunolocalization) are needed to locate the transcriptionally active ribosomal loci at the secondary constrictions.	Terasaka and Tanaka, 1974; Gao et al., 2012; Gürdal and Özhatay, 2018
All SAT-chromosomes are satellited chromosomes	SAT-chromosome is not a synonym for satellited-chromosome, but implies either a satellited chromosome or a chromosome with a secondary constriction associated with the formation of the nucleolus, which does not have a satellite.	Berger, 1940
Only satellited chromosomes show NOR loci	Active 35S rDNA loci may be present at the terminal ends of chromosomes.	Battaglia, 1955; Rosato et al., 2017; Fehrer et al., 2021
All ribosomal loci are NOR	Only transcriptionally active 35S rDNA loci (which are evaluated through silver staining, the immunolocalization of histone methylation or histone deacetylation, and DNA cytosine methylation) involved in the formation of the nucleolus are NORs.	Poczai and Hyvönen, 2010; Marques et al., 2011; Milioto et al., 2019
Ribosomal loci detected by FISH are NOR loci	Hybridization *in situ* techniques using radioactive or non-isotopic probes detect all 35S rDNA loci above a threshold of the number of repeats, irrespective of their transcriptional activity.	Cuñado et al., 2000; Abd El-Twab and Kondo, 2010; Milioto et al., 2019
The number of ribosomal loci can be inferred from the number of satellite-bearing chromosomes	Only active rDNA loci are located at secondary constrictions. Silenced and pseudogene loci are not transcribed and cannot be seen as decondensed chromatin near the satellite body.	Galián et al., 2012; Rosato et al., 2017; Báez et al., 2020
FISH signals using 35S rDNA probes always shows canonical ribosomal loci	The differential amplification of coding and spacer sequences of the rDNA cistron and their transposition to other chromosomes has been reported in several species. These sites can be detected as FISH signals if significant similarity exist between the DNA probes used and the target sequences, but they are not true canonical rDNA loci.	Maggini et al., 1991; Guimond and Moss, 1999; Macas et al., 2003

**TABLE 2:** Patterns of intraindividual 35S rDNA and NOR variation in plants.

Assumption	Comments	Selected references
The number of 35S rDNA loci is constant between plant tissues from a single individual	Aberrant mitosis in the binucleate tapetal cells of some organisms results in rDNA instability regarding the number of locus within a single tissue.	Chiavarino et al., 2000; Mursalimov and Deineko, 2018
The number of NOR loci is invariant within individuals	The differential suppressing of duplicated NOR loci by epigenetic silencing (differential amphiplasty or nucleolar dominance) may differ between tissues of a single individual. Reports indicating combined uniparental and biparental tissue-specific expression are known.	Dobešová et al., 2015; Sochorová et al., 2017; Borowska-Zuchowska et al., 2021
The number of ribosomal loci is not affected by vegetative propagation	Tissue culture by *in vitro* techniques has been reported to induce drastic changes (somaclonal variation) regarding the number of repeats and loci in many species.	Bairu et al., 2011; Rosato et al., 2016b
Ribosomal loci are always located in chromosomes of the regular complement	B-chromosomes can show silenced or active rDNA sites in some species. Individual variation in such accessory chromosomes may result in contrasting numbers of ribosomal loci.	Jones et al., 2008; Houben et al., 2013; D’Ambrosio et al., 2017
Ribosomal loci are always present in autosome chromosomes	The presence of 35S rDNA loci have has also been reported in sexual chromosomes.	Nakayama et al., 2001; Fujisawa et al., 2003; Nakao et al., 2005; Deng et al., 2012
The number of NOR sites are not gender dependent in dioecious species	In addition to their presence in autosomes, 35S rDNA loci may be linked to sexual chromosomes. The differential silencing of transcriptional activity in individual sites is known for the male individuals of some species. In these cases, male and female plants differ in the overall number of secondary constrictions and Ag-NOR sites.	Nakao et al., 2005

**TABLE 3:** Evolutionary trends in the number and activity of rDNA and NOR loci: cautions and limitations.

Assumption	Comments	Selected references
Diploid species are characterized by the presence of a single 35 rDNA locus	It has been estimated that about 65% of the analyzed diploid species of spermatophytes show two or more 35S rDNA loci as assessed by FISH.	Roa and Guerra, 2012; García et al., 2017
The presence of a single NOR locus is the evolutionary derived state for seed plant lineages	Most data available for the number of NORs in plants have not been discussed against phylogenetic inferences. This precludes the building of solid hypotheses about patterns of rDNA site change and the identifying the ancestral and evolutionary derived states.	Díaz Lifante, 1996; Bisht et al., 1998
Within closely related lineages the number of satellited chromosomes is associated to the ploidy level of the species	The number of satellited chromosomes may vary between congeneric species showing the same ploidy level.	Díaz Lifante, 1996; Bisht et al., 1998
An increase in the number of rDNA loci is always linked to polyploidy	The amplification of ribosomal loci may take place within homoploid lineages in the absence of polyploidy by means of transposition, chromosomal translocations and disploidy.	Hidalgo et al., 2017; Rosato et al., 2017; Totta et al., 2017
Within lineages the ancestral number of 35S rDNA loci is usually one	The ancestral number of rDNA loci is variable between lineages. Dynamic and complex changes have been documented in their evolutionary history regarding the amplification and deletion of repeats and loci involving chromosome repatterning.	Ran et al., 2001; Mishima et al., 2002; Weiss-Schneeweiss et al., 2008; Rosato et al., 2015; Totta et al., 2017
The number, genomic location and activity of 35S rDNA loci are constant within species	Changes in the number of loci and sites, their chromosomal position and the number of repeats per locus have been detected in several species, even within populations.	Schubert, 1984; Schubert and Wobus, 1985; Weiss-Schneeweiss et al., 2013; Rosato et al., 2017

**TABLE 4:** Intra- and interindividual variation in nucleoli number: assumptions about their use in plant evolution.

Assumption	Comments	Selected references
The number of nucleoli in interphase nuclei equates to the number of ribosomal loci	Only the transcriptionally active rDNA loci give rise to nucleoli. If active and inactive 35S rDNA loci are present in a species the number of nucleoli will be formed only by the active loci. Since nucleoli tend to fuse (mononucleolation) during the cell cycle, only the highest number of nucleoli detected should be taken as the number of NORs present in the chromosome complement.	Brasileiro-Vidal et al., 2003; Rosato et al., 2016b
The number of nucleoli is constant within all tissues of a single plant	Cytomixis (the migration of nuclei and their components, including nucleoli, between two cells) has been reported in several plant tissues. This could lead to the observation of different numbers of nucleoli, which may differ also in dimensions, because of intercellular migration. In hybrid and allopolyploid species, the number of nucleoli may vary between tissues (usually between somatic and reproductive tissues) due to nucleolar dominance, the uniparental expression of the 35S rDNA genes.	Mursalimov and Deineko, 2011, 2018; Kumar and Singhal, 2016 Hasterok and Maluszynska, 2000; Idziak and Hasterok, 2008; Borowska-Zuchowska and Hasterok, 2017
The number of nucleoli can be used as an alternative way to determine the ploidy level	Active rDNA loci in hybrids and allopolyploids can be transcriptionally silenced by epigenetic processes (nucleolar dominance). Thus, nucleolar suppression may lower the expected number of nucleoli in polyploids. In addition, the deletion of rDNA units in duplicate loci may eventually lead to the elimination of entire NOR loci and the associated nucleoli.	Pikaard, 2000a; González-Melendi et al., 2005; Rosato and Rosselló, 2009

This paper aims to discuss the limitations of nuclear 35S rDNA markers (i.e., site number, transcriptional activity, nucleolar dominance) based on cytological and karyological experimental work to draw sound biological and evolutionary conclusions in a systematic and phylogenetic context. Also, we provide clarification for some conceptual misconceptions usually found in published work that could help lead to an insightful utilization of the ribosomal world in plant evolution.

## The Nuclear 35S rDNA Locus

### Terminology: A Primer of Confusion

**FIGURE 1:**
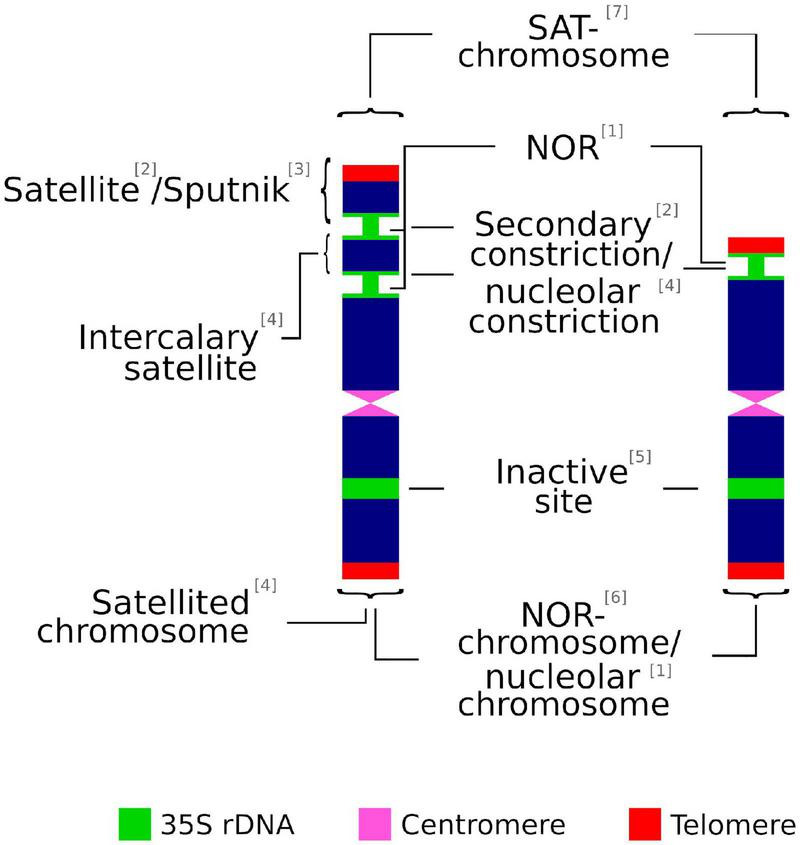
Graphical summary of terms used to describe the chromosomic domains associated to the 35S rDNA locus. (1) McClintock, 1934; (2) Darlington, 1926; (3) Battaglia, 1999; (4) Battaglia, 1955; (5) Leitch and Heslop-Harrison, 1993; (6) Schwarzacher and Wachtler, 1983; (7) Heitz, 1931.

Most of the following terms are often used interchangeably in scientific literature: secondary constriction, nucleolar constriction, satellite, sputnik, intercalary satellite, nucleolar organizing regions (NORs), satellite(d) chromosome, SAT-chromosome, nucleolar chromosome, NOR chromosome, and 35S rDNA locus (Figure 1). However, in some cases there are significant differences that might not be rightly appreciated. Thus, inappropriate or confusing denominations may cause incorrect judgment on technically accurate data (Table 1).

The association of non-centromeric (i.e., secondary) constrictions (Darlington, 1926) and nucleolus formation during the interphase was noted early on by Heitz (1931) and McClintock (1934). The latter is credited for coining the term nucleolar organizer (later changed to NOR) to describe the chromosome region in *Zea mays* that was involved in the formation of the nucleolus (Pikaard, 2000a). In addition, a heterochromatic knob of the chromosome, the satellite, is present at the telomeric-proximal site of the secondary constriction, but is not involved in the formation of the nucleolus (Chen et al., 2000).

The term satellite chromosome is commonly used in the same way as SAT-chromosome. However, this is a misinterpretation recognized early on by Berger (1940), whose efforts to distinguish both technical terms have been repeatedly ignored in cytological literature (Battaglia, 1955). SAT was coined by Heitz (1931) as an abbreviation of Sine Acido Thymonucleinico (Without Thymonucleic Acid, the early denomination of DNA) and it refers to secondary constrictions. The decondensed chromatin at the NOR observed by Heitz (1931) showed less stained intensity (achromatic) than other chromosomal regions and was wrongly interpreted as lacking DNA. Accordingly, and following the original meaning, SAT-chromosome is not a synonym for satellited-chromosome but implies either a satellited chromosome or a chromosome with a secondary constriction that is associated with the formation of the nucleolus but does not have a satellite (Berger, 1940). In addition, and in contrast to the original meaning coined by McClintock (1934), some authors broadly define NOR sites as the chromosomal segments that contain ribosomal genes, irrespective of their transcriptional activity (Trerè, 2000).

In maize, there is a single pair of chromosomes showing secondary constrictions (i.e., two nucleolar chromosomes) that are also satellited chromosomes, a single active 35S rDNA locus or NOR, and a single associated nucleolus (Chen et al., 2000). This chromosomal rDNA pattern shows a complete agreement between chromatin decondensation sites (secondary constrictions), transcriptional active regions (NOR), the physical location of 35S rDNA units and the number of nucleoli formed (McClintock, 1934; Khuong and Schubert, 1985; Sadder and Weber, 2001).

**FIGURE 2:**
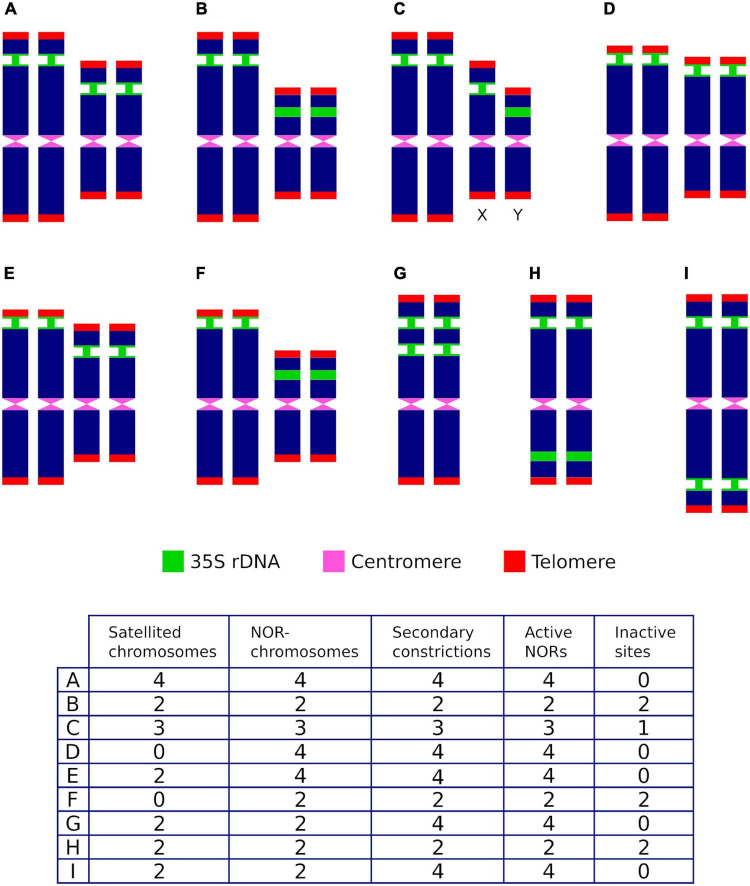
Hypothetical ribosomal phenotypes **(A–I)** and their associated ribosomal descriptors for individuals showing two rDNA loci and differing in transcriptional activity, the number of chromosome pairs where they are located, the position of the loci along the chromosome arms, the presence of sites in sexual chromosomes (X,Y), the number of secondary constrictions and the presence of satellites. Active sites are represented with narrow green stalks.

Complexity may arise in lineages, however, when mechanisms involved in altering the number of rDNA loci have occurred along their evolutionary history. These bursts of rDNA amplification include the dispersion of loci by structural chromosome rearrangements at homoploid levels as well as the transposition and amplification of rDNA copy numbers (Datson and Murray, 2006; Rosato et al., 2017), and have involved both diploid lineages and complex scenarios of ancestral and more recent polyploid events (Rosato et al., 2015). These events may have resulted in karyotypes that eventually contain more 35S rDNA sites than expected when compared to the ancestral or parental lineages. When karyotypes contain more than one 35S rDNA locus, it clearly shows that care should be exercised to describe the chromosomal rDNA pattern, since not all the commonly used terms are equivalent and should not be broadly used as mere alternatives with similar or identical meanings (Figure 2).

As an example that illustrates this issue, let us consider that the chromosomal complement of a hypothetical diploid species contains two 35S rDNA loci. After taking into account all heretofore reported relevant genomic locations and transcriptional activity alternatives that occur in the karyograms of seed plants (i.e., the number of active and silenced sites, the number of chromosome pairs where they are located, the position of the loci along the chromosome’s arms, the presence of sites in sexual chromosomes, the number of secondary constrictions and the presence of satellites), up to eight potential ribosomal phenotypes may be described (Figure 2) by common descriptors frequently used in the literature (the number of SAT-chromosomes and satellited chromosomes, secondary constrictions and NOR sites). Thus, for a given karyotype (1) the number of SAT-chromosomes, satellite(d) chromosomes and nucleolar chromosomes may differ, (2) the counting of secondary constrictions may not be predictive of the overall number of rDNA loci and satellite(d) chromosomes, (3) some 35S rDNA loci may not be transcriptionally active and are not, accordingly, NOR sites, and (4) an odd number of NOR sites does not point to positional hemizygosity and may be attained instead by epigenetic silencing.

### The Cytological Recognition of 35S rDNA Loci

**FIGURE 3:**
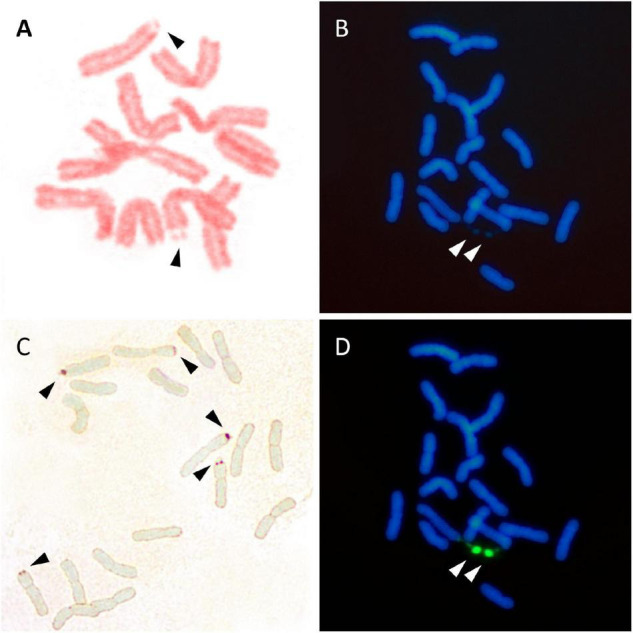
Visualization of 35S rDNA locus using conventional staining **(A,B)**, silver impregnation **(C),** and FISH **(D)**. **(A)** *Lathyrus pisiformis*. A single chromosome pair with secondary constrictions after Feulgen staining. **(B)** *Achillea maritima*. The presence of two secondary constrictions (arrows) are observed using DAPI staining. **(C)** *Anacyclus homogamos*. Five NORs are present after Ag-NOR staining (arrows). **(D)** *Achillea maritima*. A single 35SrDNA locus is located on the secondary constrictions after FISH (green signals).

Several methods are currently used to physically map the 35S rDNA units in plant cells. These include (1) the observation of chromosomes with conventional stains (e.g., acetic orcein, hematoxylin, Feulgen reagent, carmin acetic acid, Giemsa C-banding) or fluorescent dyes binding the DNA, e.g., DAPI (4’,6-diamidino-2-phenylindole) and CMA (Chromomycin A3) (Figures 3A,B), looking for the presence of secondary constrictions that are associated with the nucleolus (Maluszynska et al., 1998), (2) the observation in secondary constrictions of acidic, non-histone proteins that bind silver ions (argyrophilic) and are differentially stained by silver impregnation (Goodpasture and Bloom, 1975; Tucker et al., 2010; Figure 3C), (3) the distribution of epigenetic marks at the rDNA loci such as methylation and deacetylation patterns of histone H3 and the pattern of DNA methylation (5-methylcytosine sites) (Marques et al., 2011; Borowska-Zuchowska and Hasterok, 2017), and (4) the detection of tandemly repeated rDNA copies using *in situ* hybridization techniques (Heslop-Harrison and Schwarzacher, 2011; Jiang, 2019; Figure 3D).

The above procedures differ in the level of specificity, sensitivity, and reproducibility in the required quality of the target cells, as well as in the time and complexity of the experimental work. Most importantly, the techniques differ in the type of information retrieved. The first three approaches (secondary constrictions, silver staining, and epigenetic patterns) basically identify the 35S rDNA sites which are transcriptionally active in the cell, and which may be cytologically visible from interphase to metaphase of mitosis and prophase I of meiosis. In contrast, *in situ* hybridization techniques on nuclei and chromosomes using DNA labeled probes identify both the active and inactive 35S rDNA sites. None of the available techniques are free of experimental drawbacks and limitations and the best results are usually obtained with a combination of methods.

The visualization of NORs by observing secondary constrictions using conventional and fluorochrome staining is a fast but rather crude technique which is still in use. It has been reported that the decondensation of the rDNA chromatin occurs in different ways depending on the cell type and the analyzed species (Leitch, 2000). It has even been suggested that the activity of rDNA at different loci may vary over the course of the cell cycle, with their transcription being determined in a time and region-specific manner (Li et al., 2006; Chandrasekhara et al., 2016). In addition, the length of the decondensed chromatin is connected to the number of rDNA units being transcribed. Thus, the observation of secondary constrictions on mitotic chromosomes may be difficult to obtain unless other confirmatory, more powerful methods are used. The highest level of chromatin decondensation displayed in meiosis, in contrast to mitotic chromosomes, makes prophase I (generally, from pachytene to diakinesis) an excellent stage for a highly accurate physical mapping of chromosomal landmarks (De Jong et al., 1999; Sepsi et al., 2018), including NORs. However, the association of the homologous chromosomes bearing NORs (nucleolar bivalent or NOR-bivalent) with the nucleolus may appear disconnected and is not always easily observed (McClintock, 1934) in a similar way as has been reported for somatic chromosomes. These explanations alone or in combination may be related to the unsuccessful cytological detection of secondary constrictions in plant chromosomes and should considerably mitigate their use when unorthodox observations are obtained. For instance, data on the presence of secondary constrictions in the karyotypes of the endemic flora of the Balearic Islands using conventional karyological techniques is known for only 13% of the species (Rosselló and Castro, 2008).

Nucleolar organizing regions contain argyrophilic proteins that are selectively stained by silver methods allowing their identification throughout the nucleolar area (Ag-NORs). Unfortunately, the use of such a sensitive stain is difficult to standardize and many technical improvements have been reported for plant and animal organisms from time to time (e.g., Biliński and Bilińska, 1996; Trerè, 2000). However, non-optimal silver impregnation resulting in a bright background of nuclear DNA (Goodpasture and Bloom, 1975) or unspecific results (for instance, the staining of centromeric regions; Báez et al., 2020) may compromise the reliability of the technique. Since the presence of NORs in a decondensed state is one of the prerequisites for positive silver staining (Jiménez et al., 1988), the detection of Ag-NORs is not always reliable. Research has revealed the presence of active rDNA sites which are barely visible or not reflected by secondary constrictions on metaphase chromosomes, even after Ag-NOR staining, in species from lineages that are not closely related (Berg and Greilhuber, 1993). These observations may have various origins, like a small number of active ribosomal genes, a low transcriptional activity in some tissues or a different condensation level of chromatin in the NORs.

Fluorescence *in situ* hybridization (FISH) is by far the preferred karyological technique to assess 35S rDNA loci in plant chromosomes and very thorough compilations are available online (García et al., 2017). However, this approach alone does not differentiate the active and transcriptionally silent loci, and sequential methods (previous Ag-staining or the immunolocalization of epigenetic markers followed by FISH) are needed to obtain the most comprehensive results related to the number and functionality of 35S rDNA. FISH can reveal the major 35S rDNA loci of the genome, but it also has limitations or a lack of repeatability to detect minor loci characterized by a small number of rDNA units.

### Intraindividual and Interspecific Variation in the Number and Activity of rDNA Sites: Sexual Chromosomes, Accessory Chromosomes, and Nucleolar Dominance

Assessments have reported that the number of secondary constrictions is gender dependent in several gymnosperm species, as *Ephedra foliata* (Mehra and Khitha, 1981), *Cycas* sp. pl. (Abraham and Mathew, 1962; Sangduen et al., 2007, 2009), *Ginkgo biloba* (Nakao et al., 2005), and *Stangeria eriopus* (Kokubugata et al., 2002). Observations in *Ephedra* and *Cycas* were based on conventional cytological techniques suggesting that faint satellites located at the end of the postulated sexual chromosomes (XY system), differentiate between X (with a satellite at each arm end) and Y chromosomes (a satellite at one end only, or none). Sangduen et al. (2009) concluded that the karyotype of male and female *Cycas* species could be clearly distinguished by the number of homomorphic and heteromorphic chromosome pairs possessing secondary constrictions. Although uncertainties about the accuracy of these cytological observations have been expressed by some authors dealing with sex chromosomes in gymnosperms (Ming et al., 2011), no available experimental work has been produced to dispute these findings, some of which were corroborated in somatic and generative tissues (Abraham and Mathew, 1962). Two heteromorphic chromosome pairs connected to rDNA sites were detected in *Stangeria* using a homologous probe (Kokubugata et al., 2002). The failure to associate this finding with sex chromosomes was due to the fact that the sex of the individual investigated was unknown.

Similar observations were reported in *G. biloba* chromosomes, where male individuals showed three chromosomes with secondary constrictions whereas four were present in female plants (Nakao et al., 2005). Additional studies by Lan et al. (2008) reported that in male individuals, the satellites of chromosome 1 (the biggest of the complement) were homomorphic, while in females they were heteromorphic, and one appeared to be bigger than the other. Both male and female plants showed the same number of rDNA sites (four) when performing FISH using a homologous rDNA probe (Nakao et al., 2005). Therefore, the differential epigenetic silencing of a whole rDNA site associated to the presence of a different rDNA copy number in homologous chromosomes may explain these cytological singularities in *Ginkgo*.

Accessory or supernumerary B chromosomes (Bs) are one of the most captivating topics of the evolution of the nuclear eukaryote genome. Bs are additional dispensable genetic components that contribute as part of the genome of a great diversity of organisms including plants (D’Ambrosio et al., 2017; Houben et al., 2019; Pokorná and Reifová, 2021). Several studies have revealed the lack of essential genes in their composition, except for the eventual presence of 35S and 5S rDNA families (Sýkorová et al., 2003; Jones et al., 2008; Houben et al., 2013). The activity of 35S rDNA sites located on Bs have been analyzed, and diversity in the transcriptional activity has been revealed in a similar way as is known for the chromosomes of the regular chromosomal complement (A chromosomes).

In *Brachyscome dichromosomatica* the 35S rDNA sites located at the large and micro Bs are silenced and are not associated to the nucleolus (Marschner et al., 2007). In contrast, in *Plantago lagopus* and *Crepis capillaris* the Bs are transcriptionally active (NOR) and contribute to the genesis of the nucleolus (Jones, 1995; Dhar et al., 2002). In *Nierembergia aristata*, Bs possess not only strong nucleolar activity, but also show nucleolar competition with the A chromosomes (Acosta and Moscone, 2011). This phenomenon could be analogous to the nucleolar dominance that occurs in interspecific hybrids (see below). Moreover, *Secale cereale* show the presence of B chromosomes without 35S rDNA sites. Interestingly, it has been suggested that Bs changes the rDNA organization pattern in interphase nuclei as detected by a drastic increase of rDNA condensed blocks inside the nucleolus (Delgado et al., 2004). Available evidence suggests that the rDNA alteration is caused by the presence of the B chromosomes themselves rather than by an obvious dosage effect (Delgado et al., 2004). The singular nature of B chromosomes exhibiting specific genomic features illustrates the need for caution when analyzing the pattern of nucleolar activity (Table 2).

Differential amphiplasty, also known as nucleolar dominance or reversible NOR silencing, is a conspicuous cytological and complex molecular phenomenon which was known to early plant cytogeneticists (Navashin, 1934). When the nuclear genomes of related species are merged by hybridization processes their NORs may differ in their competitive ability to transcribe the ribosomal genes and form the nucleolus. The net result of this is that a set of NORs from one of the parental species is epigenetically suppressed and fails to form secondary constrictions, thus leading to a decrease in the number of nucleoli. Accordingly, the NORs of hybrid and allopolyploids may not be added together and thus fail to reveal all the active rDNA sites inherited from the two parents (Tucker et al., 2010).

Interestingly, in individuals of hybrid origin, the presence of nucleolar dominance may be tissue-specific and the rDNA sites could be differentially expressed (Chen and Pikaard, 1997; Borowska-Zuchowska et al., 2021). These combined uniparental and biparental patterns of NOR silencing showing a contrasting number of NORs has detected in vegetative and reproductive tissues. In allotetraploid *Brassica* species (*B. carinata*, *B. juncea*, *B. napus*), rRNA genes silenced in leaves were found to be transcribed in all floral organs where biparental gene expression was maintained (Chen and Pikaard, 1997; Sochorová et al., 2017). However, Hasterok and Maluszynska (2000) reported that nucleolar dominance did not occur in root tip cells from these polyploid species. Biparental rDNA expression was found in roots, flowers and callus in the allotetraploid *Tragopogon mirus* (Dobešová et al., 2015). However, uniparental dominance was maintained in its leaves. These observations clearly indicate that silenced and derepressed rRNA genes may occur not only during developmental stages, but within vegetative and reproductive tissues. Since cytogenetic observations are preferentially limited to favorable cell types such as root tips and pollen mother cells, there may be contrasting reports on the number of active NOR sites (Table 2).

### Ribosomal Loci as Evolutionary Markers: Some Cautions on Premises and Interpretations

Detailed knowledge of the number of rDNA loci, their genomic location and the linkage of the 35S and 5S rDNA units has been assessed for a substantial number of species (García et al., 2012, 2014; Roa and Guerra, 2012; Vitales et al., 2017). In contrast, compilations of NOR activity are very few and, unfortunately, this knowledge has not been updated since the work of Lima-de-Faria (1976). In addition, most information on the dynamics of rDNA loci variation has not been thoroughly analyzed under explicit phylogenetic frameworks, and the data has been subject to speculative interpretations. In sharp contrast with what is known in selected polyploid species, the evolutionary patterns of rDNA loci number in predominantly diploid lineages are insufficiently understood and have received less attention (e.g., Datson and Murray, 2006; Totta et al., 2017). Moreover, most previous studies on rDNA loci changes lack explicit temporal frames, and as a result, their dynamics could not be assessed with certainty. The fact that surprisingly few studies have addressed intrapopulation, interpopulation and interspecific levels of rDNA variability in non-model wild plants, may question the assumptions of generalized evolutionary trends that are usually based on very few case studies.

Thus, the pervading perceptions that (1) the number, genomic location, and activity of 35S rDNA loci are constant within species, (2) diploid species are usually characterized by a single rDNA locus, (3) the ancestral number of 35S rDNA loci is usually one, (4) the presence of a single NOR locus is the derived state for seed plant lineages and (5) the increase in the number of rDNA loci is mostly linked to polyploidy, should all be checked on a case-by-case basis (Table 3).

The cytogenetic research conducted on the Mediterranean *Anacyclus* (Asteraceae), a diploid genus comprising nine species of weedy annuals and a few perennials, has provided relevant results illustrating how the above statements are not generally applicable in plants. Available karyological rDNA data was first obtained by Schweizer and Ehrendorfer (1976) and Ehrendorfer et al. (1977), who determined the number of active rDNA loci for all species based on a small number of accessions. Later, Rosato et al. (2017) determined the number and chromosomal position of 35S rDNA sites in 196 individuals from 47 populations in all *Anacyclus* species using FISH. The following conclusions could be firmly established from the results obtained by both research teams. First, the level of rDNA site-number variation detected within most *Anacyclus* species was outstanding and included both intra-specific and intra-population polymorphisms that encompassed a large part of the range of variation found in all angiosperms. Second, no clear association could be established between the phylogenetic position of the species and the number of rDNA sites. Third, the cytogenetic changes underlying the inferred rDNA dynamism were not related to polyploidy and were likely triggered by genomic rearrangements resulting from contemporary hybridization. Finally, the number of NORs in the genus was not associated to the phylogenetic ancestry of the species; the perennial clade showed two loci whereas the most derived annual species presented three loci.

Inferring polyploidy based on the number of NOR and rDNA sites may be misleading. An increase of NORs, and thus of nucleoli, is accomplished not only by genome duplication (Table 3), as had been earlier postulated (Gates, 1942; Fankhauser and Humphrey, 1943). Additional processes including structural rearrangements, ectopic recombination and rDNA transposition have been proposed as alternative mechanisms to explain NOR amplification within genomes (Pikaard, 2000b; Cabrero and Camacho, 2008, and references therein). Specifically, the intragenomic mobility of rRNA genes because of transposon activity, which can produce a translocation of rDNA copies to new genomic sites has been substantiated in seed plants, and it has been hypothesized that it is one of the major forces driving rDNA locus evolution in connection with the origin of new 35S rDNA sites (Dubcovsky and Dvorák, 1995; Raskina et al., 2004; Datson and Murray, 2006).

**FIGURE 4:**
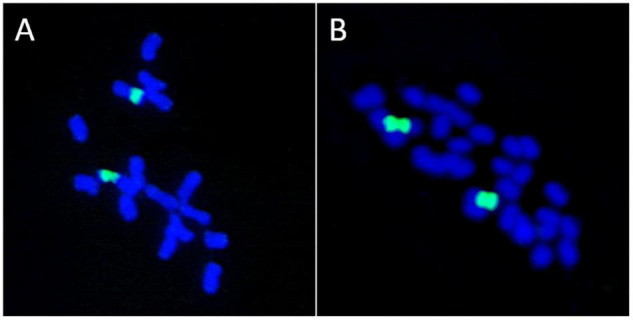
Lack of association between the number of ribosomal loci and polyploidy. **(A)** *Medicago marina*, 2*n* = 2x = 16. **(B)** *M. arborea*, 2*n* = 4x = 32. Both species possess a single 35S rDNA locus as revealed by FISH (green signals).

The basal chromosome number for each major lineage of early land plants (liverworts, mosses, and hornworts) is not known with certainty, and the topic has been greatly debated. The fact that two NOR loci were reported in some liverwort species (*Riccardia pinguis*) led Berrie (1958a,b) to hypothesize that species with *n* = 10 were polyploids derived from *n* = 5 hornwort ancestors. He further suggested that liverworts with gametophytic numbers of chromosomes of *n* = 8, 9 or 10 are basically polyploids that evolved from *n* = 10 ancestors through disploidy. The hypothesis of Berrie (1958a,b) was based on the misconception that the presence of two nucleolar chromosomes in the haploid complement of a plant is a marker of polyploidy. Recent work has shown that in bryophytes the number of 35S rDNA loci and copies are not correlated to ploidy level (Rosato et al., 2016a). Also, a lack of association between the number of 35S rDNA sites and polyploidy is known in *Medicago* (Fabaceae) where some tetraploid species (*M. arborea*, *M. strasseri*) show a single rDNA site, the same number usually present in diploid lineages (Rosato et al., 2008; Figure 4).

It is known that several independent events of entire genome duplications are characteristic of almost all fundamental lineages of land plants, and they are considered a major driving force in species diversification (Clark and Donoghue, 2018; Ren et al., 2018). Hence, attempts made to associate species with low chromosome numbers to diploid entities are confirmed to be incorrect as more and more events of paleoploidization are discovered (Clark and Donoghue, 2018). This suggests that overall assumptions connecting chromosome number and the ancestral or derived state to the number of 35S rDNA loci and NOR loci should be viewed with utmost caution. Despite these caveats, it has been estimated that 64.9–66% of the analyzed *diploid* species of seed plants show more than a single 35S rDNA locus as assessed by FISH (Roa and Guerra, 2012; García et al., 2017). These figures clearly disagree with the assumptions that the presence of a single rDNA in the genome supports the diploid status of a species.

One of the flowering plant genera that has received many efforts to infer the patterns and evolutionary significance of satellite chromosomes (referred to as SAT-chromosomes in the literature) is *Taraxacum* (dandelions), a complex genus where sexual (diploid) and, mostly, apomitic (polyploid) lineages occur. Mogie and Richards (1983) noticed the presence of two markedly divergent satellited chromosomes in the genus differing in the location and visibility of the secondary constrictions as observed after conventional Feulgen staining. The *Taraxacum*-type patterns were characterized by the presence of a conspicuous and intercalary secondary constriction, whereas the identified so-called conventional type satellite had a subterminal location and an extremely small distal euchromatic region Mogie and Richards (1983). The karyological inspection of 123 dandelion species by their SAT-chromosomes showed that the species belonging to the 10 sections hypothesized to be most primitive in the genus lack satellited chromosomes. Surprisingly, most analyzed species lacking SAT-chromosomes were reported to be sexual diploids, whereas a variable and usually unstable number of *Taraxacum*-type or conventional-type satellites was found in polyploids. Additional observations revealed a most inconsistent pattern of satellites and secondary constrictions in a single species, involving their number, size and chromosomal location, even within individuals (Richards, 1989). The variation in these cytological rDNA markers did not appear to be associated with the geographic origin of the populations sampled or the taxonomic adscription of the species, and accordingly, a constant number of satellites was rarely recorded for any species (Den Nijs et al., 1978; Krahulcová, 1993). It was suggested that such uncertainties regarding secondary constrictions might partially be explained by the experimental vagueness associated to the lack of strict uniformity of the pretreatment procedures (Den Nijs et al., 1978). Despite these concerns, however, there has been an increase in the number of reports on the number, size and morphology of satellited chromosomes for taxonomic and evolutionary purposes in *Taraxacum* (e.g., Gürdal and Özhatay, 2018; Watanabe et al., 2021). Again, the apparent lack of secondary constrictions continues to be reported in some species (*T. scaturiginosum*) (Gürdal and Özhatay, 2018). A recent study investigated the position and number of 35S rDNA loci in 38 Taraxacum species covering different reproduction modes, geographical regions and putative phylogenetic groups using FISH (Macháčková et al., 2021). Interestingly, these authors do not support the presence of the previously differentiated *Taraxacum*-type and conventional-type satellites by Mogie and Richards (1983). Most importantly, all analyzed species showed at least one secondary constriction, refuting the view that the lack of SAT-chromosomes is a reliable karyological marker in dandelions, as previously indicated (Mogie and Richards, 1983; Gürdal and Özhatay, 2018). However, the work of Macháčková et al. (2021) raises additional questions. These authors reported and provided images of the presence of conspicuous secondary constrictions in chromosomes lacking FISH signals for 35S rDNA. These findings are surprising and require additional verification to use nucleolar chromosomes as a meaningful karyological and evolutionary marker in *Taraxacum*.

The location and number of secondary constrictions have also been used as relevant cytogenetic features to investigate the karyotype evolution in the Lilieae tribe, as assessed by conventional staining (Gao et al., 2012). These authors characterized the *Notholirion* genus by the absence of nucleolar constrictions in the three analyzed species, in contrast with the recognized presence in all species of the related *Cardiocrinum*, *Fritillaria*, *Lilium*, and *Nomocharis* genera (Gao et al., 2012). Since *Notholirion* showed a basal position in the inferred phylogenetic tree based on nuclear ribosomal Internal Transcribed Spacer sequences, Gao et al. (2012) hypothesized that the lack of secondary constrictions was the ancestral state in Lilieae and that they *emerged and evolved* as the apparition of genera took place over time. Since the secondary constrictions on chromosomes represent the expression of rRNA genes which were transcribed during the preceding interphase (Tucker et al., 2010), species in the Lilieae tribe for which no nucleolar constrictions were recorded by Gao et al. (2012) should obviously present at least one chromosome pair showing active rDNA units. Clearly, the evolutionary interpretations drawn from the data appear unreliable, and new research using FISH is imperative to assess the pattern of NOR evolution in the group.

## The Nucleolus

The nucleolus is a conspicuous domain delimiting the nuclear territory of transcriptionally active and mostly de-condensed ribosomal 35S rRNA genes, where the ribosomal units are assembled (Bersaglieri and Santoro, 2019). Nucleoli can be easily observed and analyzed in interphase nuclei (also in meiotic prophase I) by the same conventional silver nitrate staining and protocols that are applied to NOR loci (Lacadena et al., 1984; Xu and Earle, 1996). In recent years, several *in situ* markers of plant nucleoli have been implemented which involve methods for tagging specific nucleolar proteins with fluorescent tags, by raising antibodies or through nascent DNA and RNA using the so-called click iT chemistry (Dvořáčková and Fajkus, 2018). Nevertheless, silver staining is a relatively easy and fast approach that is still in use and advantageous over many more demanding, expensive and time-consuming protocols. In addition, silver staining allows sequential FISH to be done in the same slides, which reveals the number, location and transcriptional activity of the 35S rDNA loci (e.g., Castro et al., 2018). Observing the nucleoli in interphase nuclei has its own value and may complement the information gained by analyzing the 35S rDNA loci in metaphase. However, care should be exercised when nucleolus descriptors (usually the number) are used as evolutionary markers in plants (Table 4) since nucleoli are dynamic substructures regarding their activity, size, position, and number (Leitch, 2000) and their morphology can be modified when several types of stress occur (Hayashi and Matsunaga, 2019).

The number of nucleoli has been correlated with the number of NORs present in the chromosome complement and in the ploidy level (Venkatesh et al., 2019). However, earlier claims suggesting a close connection (i.e., linearity) between the number of nucleoli and polyploidy (Gates, 1942; Fankhauser and Humphrey, 1943) are certainly not universally used. Thus, ploidy is not related to the nucleolar number but to the nucleolar size in the fruit cells of *Solanum lycopersicum* (Bourdon et al., 2012).

**FIGURE 5:**
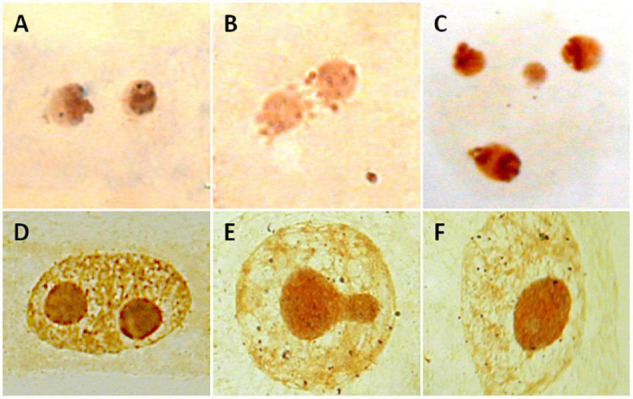
Nucleoli in interphase nuclei of *Medicago* species **(A–C)** and *Cistus heterophyllus* **(D–F)** after Ag-NOR staining. **(A)** Two nucleoli are observed in diploid *M. marina* (2n = 2x = 16). **(B)** A single nucleoli is observed in *M. arborea* (2*n* = 4x = 32). **(C)** Four nucleoli are shown in hexaploid *M. citrina* (2*n* = 6x = 48). **(D–F)** Process of mononucleation (nucleoli fusion) in *C. heterophyllus.*

Counting nucleoli is one of the techniques used to estimate the ploidy level of individuals without having dividing cells, which may be useful when using tissues with a low mitotic index (Ochatt and Seguí-Simarro, 2021). Since nucleolar fusion frequently occurs during the cell cycle (mononucleation), only the highest number of nucleoli detected should be taken as evidence of the number of active rDNA loci (Ochatt and Seguí-Simarro, 2021; Figures 5A–C). A confident determination of the number of nucleoli should be estimated in a large sample size on this basis. Nucleoli have been detected in fossilized stems of a royal fern (Osmundaceae) dating back 180 million years (Bomfleur et al., 2014) and in herbarium specimens (Laane and Höiland, 1986), paving the way to assess the estimation of ploidy levels in extinct species and populations of well-preserved museum specimens.

The assumption that the number of nucleoli is constant within individuals and species is risky and should be validated on a case-by-case basis. The variability of NOR activity, expressed as the number and size of silver-stained active sites, has been detected between cells of single individuals, and also between individuals of the same population in cock’s-foot (Besendorfer et al., 2002). In addition, atypical nucleolar behaviors across the cell cycle, including the presence of numerous small silver-positive bodies in nuclei and cytoplasm, have been reported in some species and may also compromise the interpretation of the results (Dagne and Heneen, 1992). Moreover, the migration of nuclei, including nucleoli, between plant cells (cytomixis) is a cellular phenomenon frequently observable in the male meiosis of higher plants, but the causes and consequences of cytomixis are still not entirely understood (Mursalimov and Deineko, 2018). This intercellular migration could lead to the detection of a different number of nucleoli in cells associated with the production of pollen. Finally, in hybrid and allopolyploid species, the number of nucleoli may vary between tissues (usually between vegetative and reproductive tissues and at several developmental stages) due to nucleolar dominance, the uniparental expression of the NORs (Chen and Pikaard, 1997; Hasterok and Maluszynska, 2000; Dobešová et al., 2015) as indicated above.

Additional inconsistencies related to a lack of full correspondence between the number of NORs present in the chromosome complement and the number of nucleoli have been noted. For several species, there have been reports indicating that the number of Ag-NORs in metaphase may disagree with the maximum number of nucleoli recorded in interphase nuclei (e.g., Scaldaferro et al., 2016). In *Solanum lycopersicum* meiosis, pachytene chromosomes show five 35S rDNA loci as revealed by FISH (Xu and Earle, 1996). However, only the major rDNA locus is associated to the nucleolus by Ag-NOR staining, whereas the remaining four minor loci, which are also active after silver impregnation, are not. Thus, in this species there is a correspondence between the number of Ag-NOR pachytene chromosomes and the maximum number of nucleoli observed (five), but not all NOR-bivalents could be associated to a nucleolus (Xu and Earle, 1996).

Several hypotheses attempt to explain this fact, including nucleolar association, the merging of the nucleolus during the interphase (Lacadena et al., 1984), or the occurrence of interchromosomal nucleolar dominance, where NORs from different pairs of chromosomes compete to make up the nucleoli (Tapia-Pastrana, 2020). The presence of cryptic NORs, chromosome regions apparently lacking rDNA loci, as revealed by FISH and silver staining, but which give rise to small nucleoli in interphase has been reported in plant and animal species (Sato et al., 1981; Cabrero and Camacho, 2008).

The biological reasons underlying the unsteadiness in the number of chromosomes with NOR sites and the number of nucleoli may be varied and are not fully understood. However, past and ongoing interspecific hybridization has been suggested as one of the most outstanding causes involved in the generation of cytological abnormalities that modify the regulatory system of the cell and contribute to decreasing the connection between the number of NORs and nucleoli (Levin, 1973; Tapia-Pastrana, 2020). For instance, in *Phlox hybrida* a high and significant correlation between the population hybridity and incidence of accessory nucleoli was detected (Levin, 1973). Supernumerary nucleoli were observed in *Crotalaria agatiflora* and their presence was attributed to the hybrid origin of this species (Verma and Raina, 1981). However, hybridization was rejected as the driving factor involved in the presence of accessory nucleoli in the diploid species *Trigonella foenum-graecum* although no alternative hypothesis was substantiated (Lakshmi and Raghavaiah, 1984).

Lack of association between number of nucleoli and polyploidy was detected in *Medicago* species (Rosato and Rosselló, 2009). A single nucleolus was present both in diploid (*M. marina*; 2n = 16) and tetraploid species (*M. arborea*, *M. strasseri;* 2n = 32). In addition, hexaploid *M. citrina* (2n = 48), for which the presence of three nucleoli should be theoretically expected, showed only two nucleoli (Rosato and Rosselló, 2009; Figures 5D–F). Inconsistencies also apply to non-flowering plants like bryophytes. Populations showing one or two nucleoli were registered in the gametophytic haploid (*n* = 9) *Pellia endiviifolia* noted (Newton, 1988). In contrast, a single nucleolus was present in diploid (*n* = 18) *P. borealis* (Newton, 1986).

## Author Contributions

JR: conceptualization, writing—original draft preparation, and funding acquisition. AM, MR, and JR: writing–review and editing. MR: supervision. All authors have equally contributed, read and agreed to the published version of the manuscript.

## Conflict of Interest

The authors declare that the research was conducted in the absence of any commercial or financial relationships that could be construed as a potential conflict of interest.

## Publisher’s Note

All claims expressed in this article are solely those of the authors and do not necessarily represent those of their affiliated organizations, or those of the publisher, the editors and the reviewers. Any product that may be evaluated in this article, or claim that may be made by its manufacturer, is not guaranteed or endorsed by the publisher.
